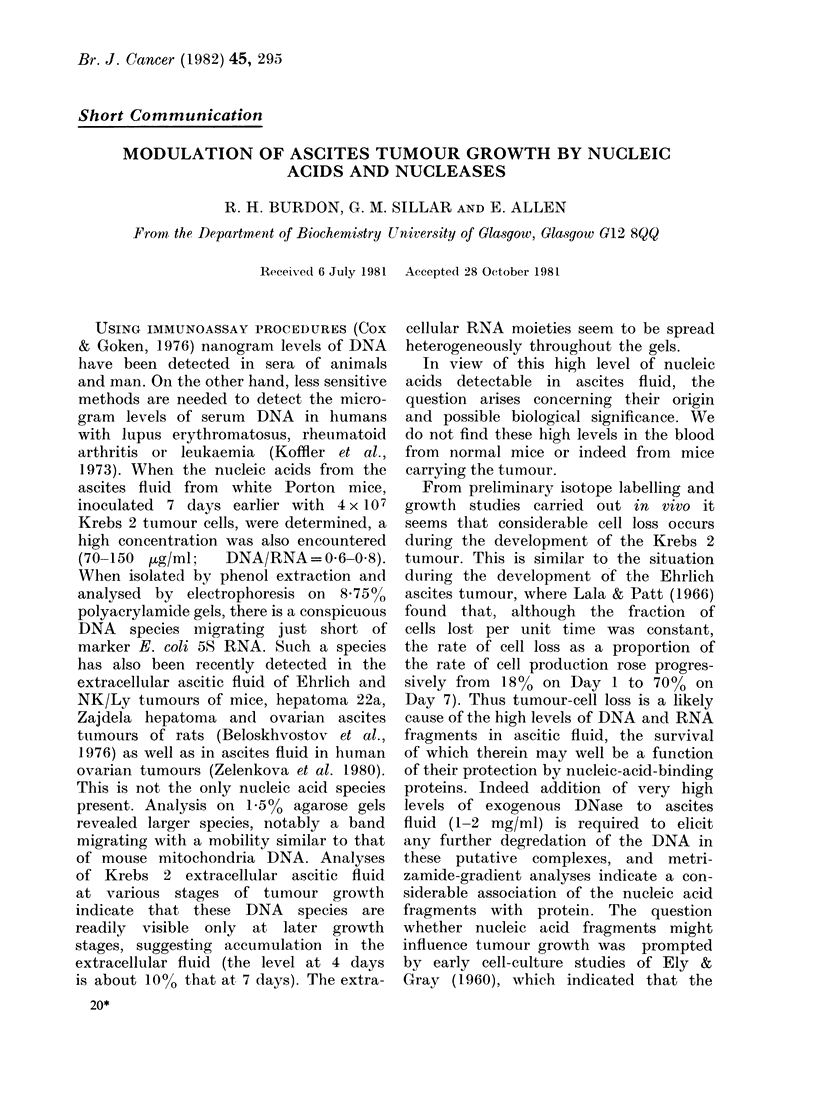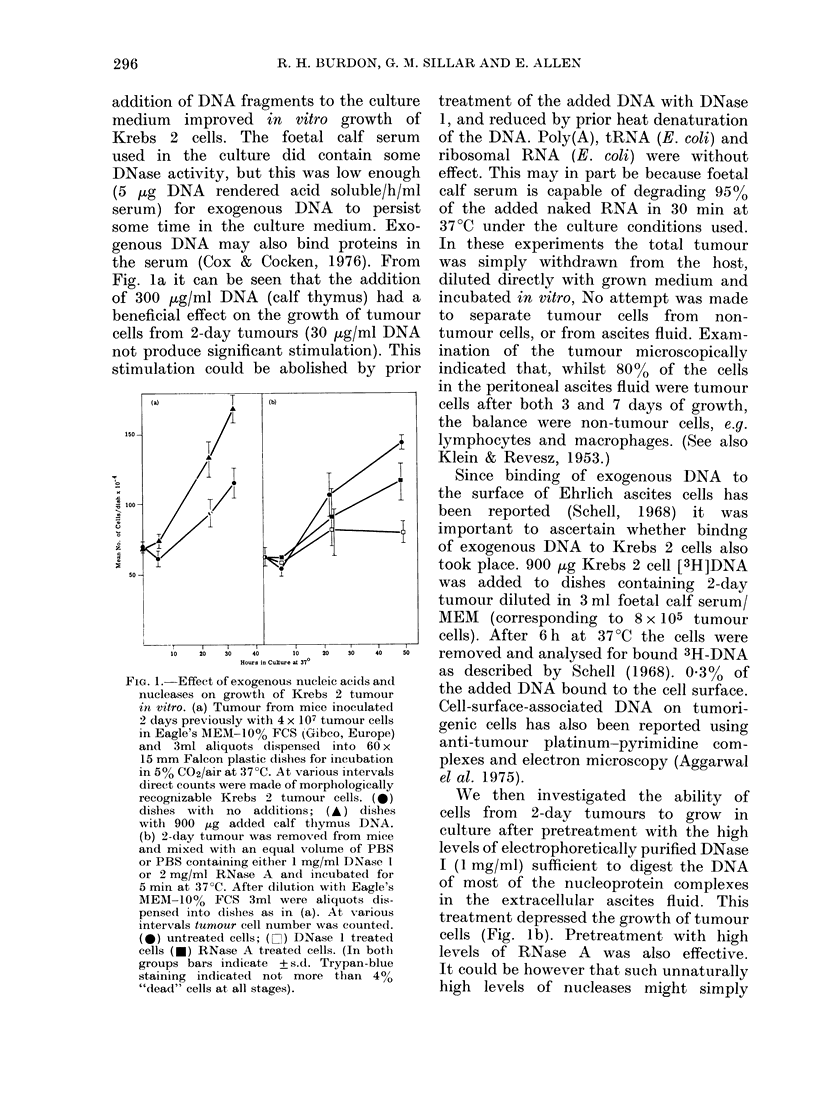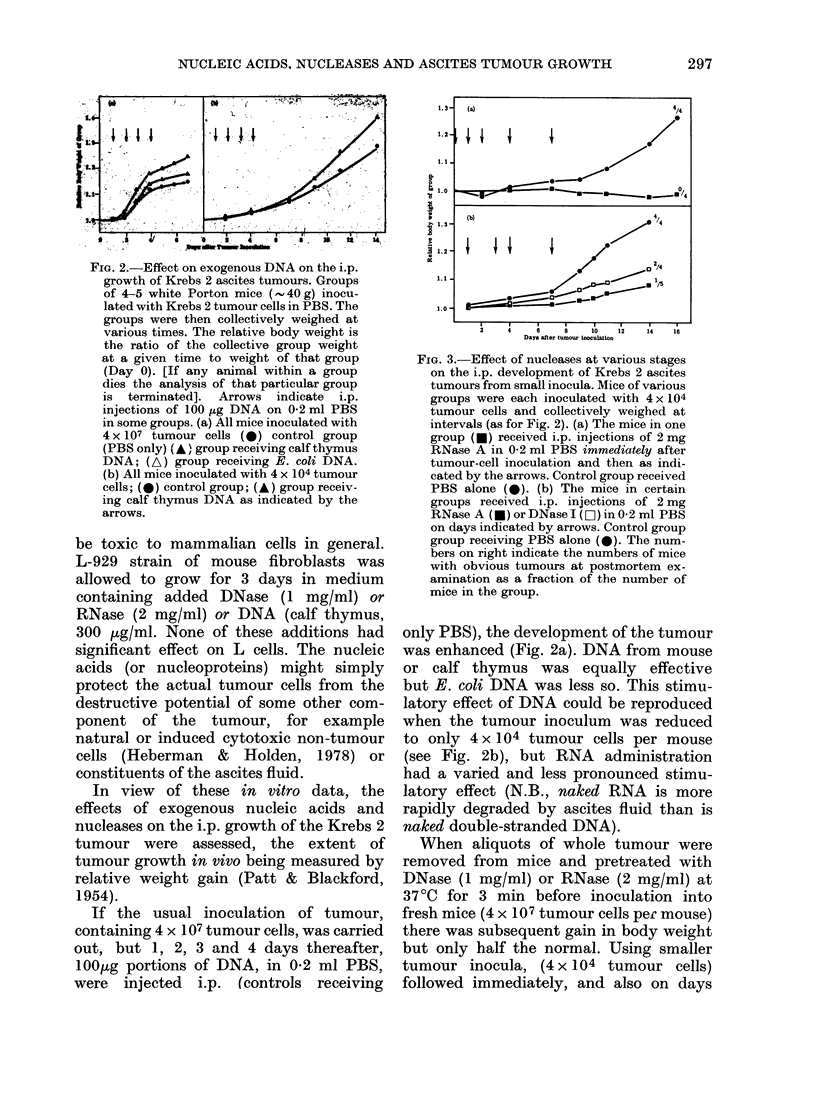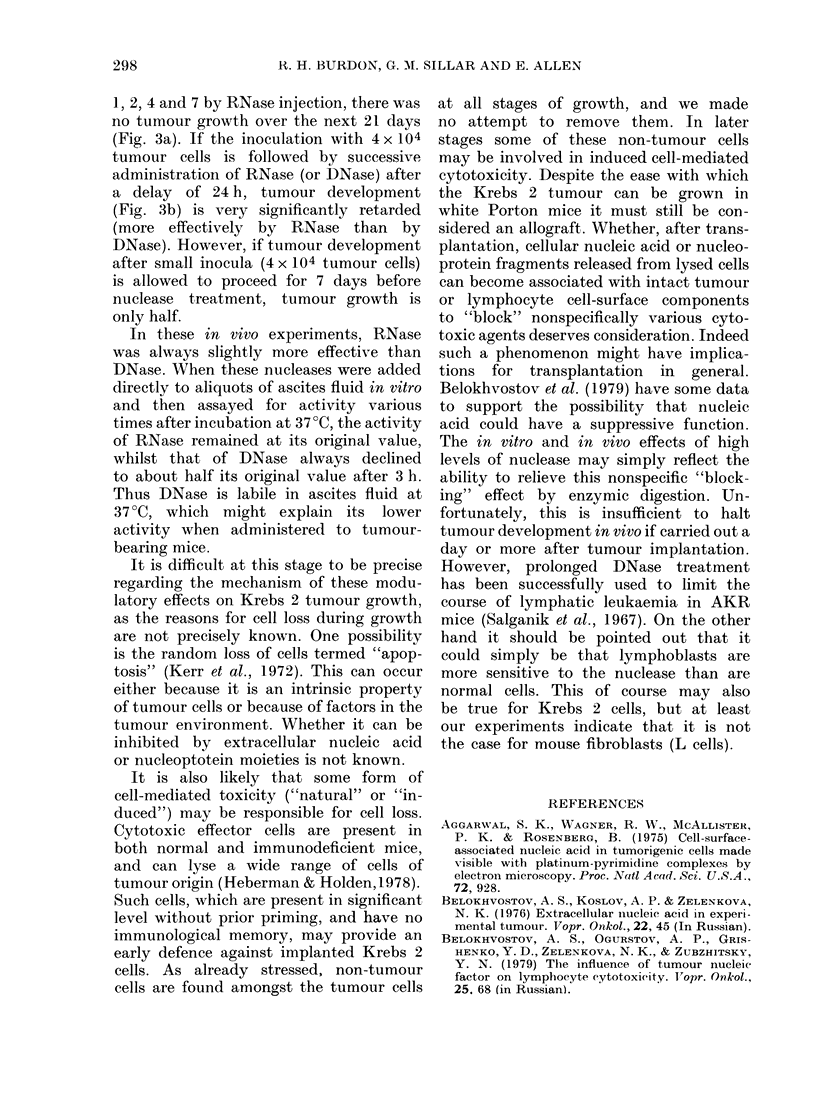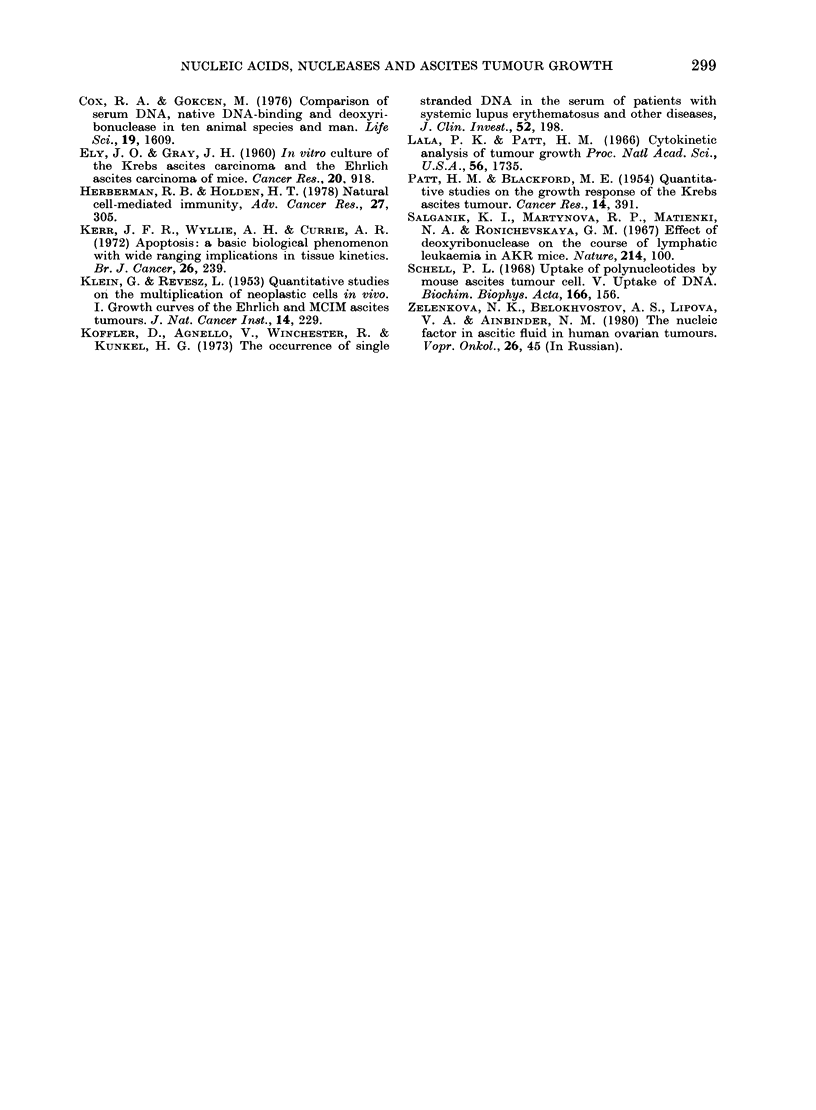# Modulation of ascites tumour growth by nucleic acids and nucleases.

**DOI:** 10.1038/bjc.1982.46

**Published:** 1982-02

**Authors:** R. H. Burdon, G. M. Sillar, E. Allen


*Br. J. Cancer* (1982) **45**, 295

***Short Communication***

### MODULATION OF ASCITES TUMOUR GROWTH BY NUCLEIC ACIDS AND NUCLEASES

R. H. BURDON, G. M. SILLAR AND E. ALLEN

*From the Department of Biochemistry University of Glasgow, Glasgow G12 8QQ*

Received 6 July 1981 Accepted 28 October 1981

USING IMMUNOASSAY PROCEDURES (Cox & Goken, 1976) nanogram levels of DNA have been detected in sera of animals and man. On the other hand, less sensitive methods are needed to detect the microgram levels of serum DNA in humans with lupus erythromatosus, rheumatoid arthritis or leukaemia (Koffler *et al.*, 1973). When the nucleic acids from the ascites fluid from white Porton mice, inoculated 7 days earlier with $4 \times 10^7$ Krebs 2 tumour cells, were determined, a high concentration was also encountered (70–150 $\mu$g/ml; DNA/RNA = 0·6–0·8). When isolated by phenol extraction and analysed by electrophoresis on 8·75% polyacrylamide gels, there is a conspicuous DNA species migrating just short of marker *E. coli* 5S RNA. Such a species has also been recently detected in the extracellular ascitic fluid of Ehrlich and NK/Ly tumours of mice, hepatoma 22a, Zajdela hepatoma and ovarian ascites tumours of rats (Beloskhvostov *et al.*, 1976) as well as in ascites fluid in human ovarian tumours (Zelenkova *et al.* 1980). This is not the only nucleic acid species present. Analysis on 1·5% agarose gels revealed larger species, notably a band migrating with a mobility similar to that of mouse mitochondria DNA. Analyses of Krebs 2 extracellular ascitic fluid at various stages of tumour growth indicate that these DNA species are readily visible only at later growth stages, suggesting accumulation in the extracellular fluid (the level at 4 days is about 10% that at 7 days). The extracellular RNA moieties seem to be spread heterogeneously throughout the gels.

In view of this high level of nucleic acids detectable in ascites fluid, the question arises concerning their origin and possible biological significance. We do not find these high levels in the blood from normal mice or indeed from mice carrying the tumour.

From preliminary isotope labelling and growth studies carried out *in vivo* it seems that considerable cell loss occurs during the development of the Krebs 2 tumour. This is similar to the situation during the development of the Ehrlich ascites tumour, where Lala & Patt (1966) found that, although the fraction of cells lost per unit time was constant, the rate of cell loss as a proportion of the rate of cell production rose progressively from 18% on Day 1 to 70% on Day 7). Thus tumour-cell loss is a likely cause of the high levels of DNA and RNA fragments in ascitic fluid, the survival of which therein may well be a function of their protection by nucleic-acid-binding proteins. Indeed addition of very high levels of exogenous DNase to ascites fluid (1–2 mg/ml) is required to elicit any further degredation of the DNA in these putative complexes, and metrizamide-gradient analyses indicate a considerable association of the nucleic acid fragments with protein. The question whether nucleic acid fragments might influence tumour growth was prompted by early cell-culture studies of Ely & Gray (1960), which indicated that the addition of DNA fragments to the culture medium improved *in vitro* growth of Krebs 2 cells. The foetal calf serum used in the culture did contain some DNase activity, but this was low enough (5 μg DNA rendered acid soluble/h/ml serum) for exogenous DNA to persist some time in the culture medium. Exogenous DNA may also bind proteins in the serum (Cox & Cocken, 1976). From Fig. 1a it can be seen that the addition of 300 μg/ml DNA (calf thymus) had a beneficial effect on the growth of tumour cells from 2-day tumours (30 μg/ml DNA not produce significant stimulation). This stimulation could be abolished by prior

FIG. 1.—Effect of exogenous nucleic acids and nucleases on growth of Krebs 2 tumour *in vitro*. (a) Tumour from mice inoculated 2 days previously with $4 \times 10^7$ tumour cells in Eagle's MEM–10% FCS (Gibco, Europe) and 3ml aliquots dispensed into 60 × 15 mm Falcon plastic dishes for incubation in 5% $CO_2$/air at 37°C. At various intervals direct counts were made of morphologically recognizable Krebs 2 tumour cells. (●) dishes with no additions; (▲) dishes with 900 μg added calf thymus DNA. (b) 2-day tumour was removed from mice and mixed with an equal volume of PBS or PBS containing either 1 mg/ml DNase 1 or 2 mg/ml RNase A and incubated for 5 min at 37°C. After dilution with Eagle's MEM–10% FCS 3ml were aliquots dispensed into dishes as in (a). At various intervals *tumour* cell number was counted. (●) untreated cells; (□) DNase 1 treated cells (■) RNase A treated cells. (In both groups bars indicate ± s.d. Trypan-blue staining indicated not more than 4% "dead" cells at all stages).

treatment of the added DNA with DNase 1, and reduced by prior heat denaturation of the DNA. Poly(A), tRNA (*E. coli*) and ribosomal RNA (*E. coli*) were without effect. This may in part be because foetal calf serum is capable of degrading 95% of the added naked RNA in 30 min at 37°C under the culture conditions used. In these experiments the total tumour was simply withdrawn from the host, diluted directly with grown medium and incubated *in vitro*, No attempt was made to separate tumour cells from non-tumour cells, or from ascites fluid. Examination of the tumour microscopically indicated that, whilst 80% of the cells in the peritoneal ascites fluid were tumour cells after both 3 and 7 days of growth, the balance were non-tumour cells, *e.g.* lymphocytes and macrophages. (See also Klein & Revesz, 1953.)

Since binding of exogenous DNA to the surface of Ehrlich ascites cells has been reported (Schell, 1968) it was important to ascertain whether bindng of exogenous DNA to Krebs 2 cells also took place. 900 μg Krebs 2 cell [$^3$H]DNA was added to dishes containing 2-day tumour diluted in 3 ml foetal calf serum/MEM (corresponding to $8 \times 10^5$ tumour cells). After 6 h at 37°C the cells were removed and analysed for bound $^3$H-DNA as described by Schell (1968). 0·3% of the added DNA bound to the cell surface. Cell-surface-associated DNA on tumorigenic cells has also been reported using anti-tumour platinum–pyrimidine complexes and electron microscopy (Aggarwal *el al.* 1975).

We then investigated the ability of cells from 2-day tumours to grow in culture after pretreatment with the high levels of electrophoretically purified DNase I (1 mg/ml) sufficient to digest the DNA of most of the nucleoprotein complexes in the extracellular ascites fluid. This treatment depressed the growth of tumour cells (Fig. 1b). Pretreatment with high levels of RNase A was also effective. It could be however that such unnaturally high levels of nucleases might simply

FIG. 2.—Effect on exogenous DNA on the i.p. growth of Krebs 2 ascites tumours. Groups of 4–5 white Porton mice (~40 g) inoculated with Krebs 2 tumour cells in PBS. The groups were then collectively weighed at various times. The relative body weight is the ratio of the collective group weight at a given time to weight of that group (Day 0). [If any animal within a group dies the analysis of that particular group is terminated]. Arrows indicate i.p. injections of 100 $\mu$g DNA on 0·2 ml PBS in some groups. (a) All mice inoculated with $4 \times 10^7$ tumour cells (●) control group (PBS only) (▲) group receiving calf thymus DNA; (△) group receiving *E. coli* DNA. (b) All mice inoculated with $4 \times 10^4$ tumour cells; (●) control group; (▲) group receiving calf thymus DNA as indicated by the arrows.

FIG. 3.—Effect of nucleases at various stages on the i.p. development of Krebs 2 ascites tumours from small inocula. Mice of various groups were each inoculated with $4 \times 10^4$ tumour cells and collectively weighed at intervals (as for Fig. 2). (a) The mice in one group (■) received i.p. injections of 2 mg RNase A in 0·2 ml PBS *immediately* after tumour-cell inoculation and then as indicated by the arrows. Control group received PBS alone (●). (b) The mice in certain groups received i.p. injections of 2 mg RNase A (■) or DNase I (□) in 0·2 ml PBS on days indicated by arrows. Control group group receiving PBS alone (●). The numbers on right indicate the numbers of mice with obvious tumours at postmortem examination as a fraction of the number of mice in the group.

be toxic to mammalian cells in general. L-929 strain of mouse fibroblasts was allowed to grow for 3 days in medium containing added DNase (1 mg/ml) *or* RNase (2 mg/ml) *or* DNA (calf thymus, 300 $\mu$g/ml. None of these additions had significant effect on L cells. The nucleic acids (or nucleoproteins) might simply protect the actual tumour cells from the destructive potential of some other component of the tumour, for example natural or induced cytotoxic non-tumour cells (Heberman & Holden, 1978) or constituents of the ascites fluid.

In view of these *in vitro* data, the effects of exogenous nucleic acids and nucleases on the i.p. growth of the Krebs 2 tumour were assessed, the extent of tumour growth *in vivo* being measured by relative weight gain (Patt & Blackford, 1954).

If the usual inoculation of tumour, containing $4 \times 10^7$ tumour cells, was carried out, but 1, 2, 3 and 4 days thereafter, 100$\mu$g portions of DNA, in 0·2 ml PBS, were injected i.p. (controls receiving only PBS), the development of the tumour was enhanced (Fig. 2a). DNA from mouse or calf thymus was equally effective but *E. coli* DNA was less so. This stimulatory effect of DNA could be reproduced when the tumour inoculum was reduced to only $4 \times 10^4$ tumour cells per mouse (see Fig. 2b), but RNA administration had a varied and less pronounced stimulatory effect (N.B., *naked* RNA is more rapidly degraded by ascites fluid than is *naked* double-stranded DNA).

When aliquots of whole tumour were removed from mice and pretreated with DNase (1 mg/ml) or RNase (2 mg/ml) at 37°C for 3 min before inoculation into fresh mice ($4 \times 10^7$ tumour cells per mouse) there was subsequent gain in body weight but only half the normal. Using smaller tumour inocula, ($4 \times 10^4$ tumour cells) followed immediately, and also on days

1, 2, 4 and 7 by RNase injection, there was no tumour growth over the next 21 days (Fig. 3a). If the inoculation with $4 \times 10^4$ tumour cells is followed by successive administration of RNase (or DNase) after a delay of 24 h, tumour development (Fig. 3b) is very significantly retarded (more effectively by RNase than by DNase). However, if tumour development after small inocula ($4 \times 10^4$ tumour cells) is allowed to proceed for 7 days before nuclease treatment, tumour growth is only half.

In these *in vivo* experiments, RNase was always slightly more effective than DNase. When these nucleases were added directly to aliquots of ascites fluid *in vitro* and then assayed for activity various times after incubation at 37 °C, the activity of RNase remained at its original value, whilst that of DNase always declined to about half its original value after 3 h. Thus DNase is labile in ascites fluid at 37 °C, which might explain its lower activity when administered to tumour-bearing mice.

It is difficult at this stage to be precise regarding the mechanism of these modulatory effects on Krebs 2 tumour growth, as the reasons for cell loss during growth are not precisely known. One possibility is the random loss of cells termed "apoptosis" (Kerr *et al.*, 1972). This can occur either because it is an intrinsic property of tumour cells or because of factors in the tumour environment. Whether it can be inhibited by extracellular nucleic acid or nucleoptotein moieties is not known.

It is also likely that some form of cell-mediated toxicity ("natural" or "induced") may be responsible for cell loss. Cytotoxic effector cells are present in both normal and immunodeficient mice, and can lyse a wide range of cells of tumour origin (Heberman & Holden,1978). Such cells, which are present in significant level without prior priming, and have no immunological memory, may provide an early defence against implanted Krebs 2 cells. As already stressed, non-tumour cells are found amongst the tumour cells at all stages of growth, and we made no attempt to remove them. In later stages some of these non-tumour cells may be involved in induced cell-mediated cytotoxicity. Despite the ease with which the Krebs 2 tumour can be grown in white Porton mice it must still be considered an allograft. Whether, after transplantation, cellular nucleic acid or nucleoprotein fragments released from lysed cells can become associated with intact tumour or lymphocyte cell-surface components to "block" nonspecifically various cytotoxic agents deserves consideration. Indeed such a phenomenon might have implications for transplantation in general. Belokhvostov *et al.* (1979) have some data to support the possibility that nucleic acid could have a suppressive function. The *in vitro* and *in vivo* effects of high levels of nuclease may simply reflect the ability to relieve this nonspecific "blocking" effect by enzymic digestion. Unfortunately, this is insufficient to halt tumour development *in vivo* if carried out a day or more after tumour implantation. However, prolonged DNase treatment has been successfully used to limit the course of lymphatic leukaemia in AKR mice (Salganik *et al.*, 1967). On the other hand it should be pointed out that it could simply be that lymphoblasts are more sensitive to the nuclease than are normal cells. This of course may also be true for Krebs 2 cells, but at least our experiments indicate that it is not the case for mouse fibroblasts (L cells).

### REFERENCES

AGGARWAL, S. K., WAGNER, R. W., MCALLISTER, P. K. & ROSENBERG, B. (1975) Cell-surface-associated nucleic acid in tumorigenic cells made visible with platinum-pyrimidine complexes by electron microscopy. *Proc. Natl Acad. Sci. U.S.A.*, **72**, 928.

BELOKHVOSTOV, A. S., KOSLOV, A. P. & ZELENKOVA, N. K. (1976) Extracellular nucleic acid in experimental tumour. *Vopr. Onkol.*, **22**, 45 (In Russian).

BELOKHVOSTOV, A. S., OGURSTOV, A. P., GRISHENKO, Y. D., ZELENKOVA, N. K., & ZUBZHITSKY, Y. N. (1979) The influence of tumour nucleic factor on lymphocyte cytotoxicity. *Vopr. Onkol.*, **25**, 68 (in Russian).

COX, R. A. & GOKCEN, M. (1976) Comparison of serum DNA, native DNA-binding and deoxyribonuclease in ten animal species and man. *Life Sci.*, **19**, 1609.

ELY, J. O. & GRAY, J. H. (1960) *In vitro* culture of the Krebs ascites carcinoma and the Ehrlich ascites carcinoma of mice. *Cancer Res.*, **20**, 918.

HERBERMAN, R. B. & HOLDEN, H. T. (1978) Natural cell-mediated immunity, *Adv. Cancer Res.*, **27**, 305.

KERR, J. F. R., WYLLIE, A. H. & CURRIE, A. R. (1972) Apoptosis: a basic biological phenomenon with wide ranging implications in tissue kinetics. *Br. J. Cancer*, **26**, 239.

KLEIN, G. & REVESZ, L. (1953) Quantitative studies on the multiplication of neoplastic cells *in vivo*. I. Growth curves of the Ehrlich and MCIM ascites tumours. *J. Nat. Cancer Inst.*, **14**, 229.

KOFFLER, D., AGNELLO, V., WINCHESTER, R. & KUNKEL, H. G. (1973) The occurrence of single stranded DNA in the serum of patients with systemic lupus erythematosus and other diseases, *J. Clin. Invest.*, **52**, 198.

LALA, P. K. & PATT, H. M. (1966) Cytokinetic analysis of tumour growth *Proc. Natl Acad. Sci., U.S.A.*, **56**, 1735.

PATT, H. M. & BLACKFORD, M. E. (1954) Quantitative studies on the growth response of the Krebs ascites tumour. *Cancer Res.*, **14**, 391.

SALGANIK, K. I., MARTYNOVA, R. P., MATIENKI, N. A. & RONICHEVSKAYA, G. M. (1967) Effect of deoxyribonuclease on the course of lymphatic leukaemia in AKR mice. *Nature*, **214**, 100.

SCHELL, P. L. (1968) Uptake of polynucleotides by mouse ascites tumour cell. V. Uptake of DNA. *Biochim. Biophys. Acta*, **166**, 156.

ZELENKOVA, N. K., BELOKHVOSTOV, A. S., LIPOVA, V. A. & AINBINDER, N. M. (1980) The nucleic factor in ascitic fluid in human ovarian tumours. *Vopr. Onkol.*, **26**, 45 (In Russian).